# School-based intervention to reduce anxiety in children: study protocol for a randomized controlled trial (PACES)

**DOI:** 10.1186/1745-6215-13-227

**Published:** 2012-11-27

**Authors:** Paul Stallard, Gordon Taylor, Rob Anderson, Harry Daniels, Neil Simpson, Rhiannon Phillips, Elena Skryabina

**Affiliations:** 1 Department for Health, University of Bath, 22-23 Eastwood, Bath BA2 7AY, UK; 2 Peninsula Medical School, PenTAG, Institute of Health Services Research, University of Exeter Medical School, University of Exeter, Exeter, EX2 4SG, UK; 3 Department of Education, University of Bath, 1 West North, Bath, BA2 7AY, UK; 4 Sirona Care and Health, Community Child Health Department, NHS House, Newbridge Hill, Bath, BA1 3QE, UK

## Abstract

**Background:**

Emotional problems such as anxiety and low mood in children are common, impair everyday functioning and increase the risk of severe mental health disorders in adulthood. Relatively few children with emotional health problems are identified and referred for treatment indicating the need to investigate preventive approaches.

**Methods/Design:**

The study is designed to be a pragmatic cluster randomized controlled trial evaluating the effectiveness of an efficacious school-based cognitive behavior therapy (CBT) prevention program (FRIENDS) on symptoms of anxiety and low mood in children 9 to 10 years of age. The unit of allocation is schools which are assigned to one of three conditions: school-led FRIENDS, health-led FRIENDS or treatment as usual. Assessments will be undertaken at baseline, 6 months and 12 months. The primary outcome measure is change on the Revised Child Anxiety and Depression Scale. Secondary outcome measures assess changes in self-esteem, worries, bullying and life satisfaction. An economic evaluation will be undertaken.

**Discussion:**

As of September 2011, 41 schools have been recruited and randomized. Final 12-month assessments are scheduled to be completed by May 2013.

**Trial Registration:**

ISRCTN23563048

## Background

Emotional problems in children are common with community surveys in the United States and in the United Kingdom indicating that at any one time up to 8% of children aged 5 to 16 years will fulfill DSM diagnostic criteria for a severe anxiety disorder with accompanying significant impairment. [1,2]. In addition, many more children experience severe anxiety or depressive symptoms that fall below criteria required for a formal diagnosis (that is, sub-threshold) but nonetheless have a significant impact upon everyday functioning. Studies suggest that during childhood and adolescence approximately one in five children will experience incapacitating anxiety or depression [3-5].

Anxiety and depression significantly impair everyday functioning and increase the risk of mental health disorders in adulthood [6-9]. The economic burden associated with childhood emotional disorders is not known although it is expected to be considerable [10]. Although effective psychological treatments are available, few children receive them. The UK National Mental Health Survey found that over an 18-month period only 22% of those children with significant mental health disorders received treatment from specialist child and adolescent mental health services [11]. In particular, those with emotional disorders were least likely to have contact with specialist services. The limited reach and availability of specialist treatment services alongside a policy shift toward early intervention has led to a growing interest in preventative approaches and a move from clinical to community settings such as schools.

Reviews of school-based emotional health prevention programs for primary school children have found evidence that both universal (provided to all) and targeted/indicated (provided to at-risk children) programs could be effective [12,13]. Multi-component programs (that is, programs that teach different skills such as relaxation, problem solving, and cognitive awareness) based upon a clear theoretical framework, particularly cognitive behavior therapy (CBT), and which included some parental input (for example, training/information), provide the strongest evidence of effectiveness. The particular benefits of CBT-based programs were also noted in a review of school-based anxiety prevention trials [14]. Although not formally tested, the effects of CBT programs were marginally larger than non-CBT interventions, with the median effect size for CBT programs of 0.57 indicating a moderate effect. However, there was considerable variation in effect size between studies, suggesting that although the content is important, mediating variables such as adherence to program fidelity, leader rapport, levels of participation and audience appeal are also important factors that will influence effectiveness.

Of the school-based CBT preventive programs that have been developed, the FRIENDS program appears particularly successful [15]. This was also noted by the World Health Organization, which identified FRIENDS as having strong evidence of being effective as a school-based intervention for anxiety [16]. The program addresses a number of the issues identified in the reviews highlighted above. It has a clear theoretical model, sufficient sessions, age-appropriate materials, enjoyable and fun activities, a structured leader manual with detailed session plans, standardized leader training, on-going supervision to ensure fidelity, a parent session, and weekly parent contact sheets.

In an initial randomized controlled trial (RCT) involving 489 children aged 10 to 12 years, significant post-intervention reductions in anxiety were reported following FRIENDS [17]. Trained teachers and psychologists were equally effective in delivering the program [17]. A subsequent study involving 594 children aged 10 to 13 years found significant reductions in anxiety, which were maintained at 12 months [18,19]. FRIENDS also had a positive effect upon mood, that is, a reduction in symptoms of low mood, in the high anxiety group. In terms of those with more significant problems, 85% of those in the FRIENDS group who initially scored above the clinical cut-off for anxiety and low mood were diagnosis free at 12 months compared to 31% in the comparison group. The effects appear to last with significant reductions in anxiety being maintained 3 years after FRIENDS [20]. In addition, comparison between children aged 9 and 10 years and those aged 14 to16 years showed that although both age groups benefited from FRIENDS, the younger group demonstrated the greatest changes in anxiety symptoms [21]. Although these results are promising, most trials have been undertaken by the program developers in Australia and no RCTs of FRIENDS have been undertaken in the United Kingdom.

## Methods/Design

### Aim of the study

The study has two primary aims:

1. To examine whether a school-based CBT program (FRIENDS delivered by either school or health professionals) is more effective than the usual school curriculum in reducing symptoms of anxiety and low mood in 9- and 10-year-old children (secondary prevention). Total Revised Child Anxiety and Depression Scale (RCADS) scores at baseline and 6 months will be compared for all children. A secondary analysis of the 10% of children in each arm who have the highest total RCADS scores will be undertaken to determine the effect upon the most symptomatic children.

2. To examine the effectiveness of FRIENDS in maintaining good emotional health, that is, to prevent high levels of anxiety and low mood from developing (primary prevention). Total RCADS scores at baseline and 12 months will be compared for the 90% of children who are not the most highly symptomatic.

### Design

Preventing Anxiety in Children through Education in Schools (PACES) is a pragmatic cluster randomized three-arm controlled trial: FRIENDS delivered by health staff or school staff *versus* usual school curriculum. The arms are summarized in Table 1.

**Table 1:** Arms of the Preventing Anxiety in Children through Education in Schools (PACES) randomized controlled trial

**Study Arm**	**Content**	**Leader**	**Number of staff**
Treatment as usual	Normal school curriculum	School staff	1
School-led FRIENDS	Structured CBT program	School staff	3
Health-led FRIENDS	Structured CBT program	Health professional	3

Interventions are delivered in schools to whole classes of children as part of the school curriculum. Assessments are undertaken at baseline, 6 and 12 months.

### Participants and eligibility

Interventions are provided as part of the school Personal, Social and Health Educational (PSHE) curriculum. All children in participating classes, that is, year 5 (9 and 10 years old) are eligible to participate. Children are ineligible if they are not attending school (for example, long term sickness, excluded, *etcetera*) or if they do not participate in PSHE for religious or other reasons.

### Ethical approval and consent

The study was approved by the Department for Health Research Ethics Committee at the University of Bath. Consent/assent involves three stages. First, eligible schools were provided with information about the study and interested head teachers were required to provide written confirmation that their school wished to participate. Second, information was posted to the home address of the parents of all eligible children. Parents were invited to return a form opting out of the study if they did not wish their child to complete the study assessments. Finally, children were provided with information about the study and were required to provide signed assent before completing a baseline assessment. Dual carer/child consent/assent was therefore required for assessment completion.

The on-going conduct and progress of the trial is monitored by an independently chaired Data Monitoring and Ethics Committee (DMEC) and a Trial Steering Committee (TSC).

### School recruitment

A list of 268 primary schools in Bath and North East Somerset, and in Swindon and Wiltshire was compiled from Local Authority information. Project information sheets were sent to the head teachers and meetings arranged with the 45 (17%) schools who expressed interest. Four schools did not return signed letters confirming participation before randomization and were therefore excluded. A total of 41 schools were randomized with one school withdrawing after randomization. The cohort therefore consisted of 40 schools (1,448 eligible children).

### Randomization

Allocation of schools took place once all schools had been recruited. Balance between trial arms with respect to key characteristics (school size, number of classes, numbers of children in year 5 classes, preferred term of delivery, preferred day of delivery, mixed-year 5 classes (that is, classes that were year 4 and year 5 combined and classes that were year 5 and year 6 combined) or single year 5 classes and level of educational attainment) was achieved by calculating an imbalance statistic for a large random sample of possible allocation sequences [22]. A statistician with no other involvement in the study randomly selected one sequence from a subset with the most desirable balance properties.

### Intervention

FRIENDS is a manualized cognitive behavior therapy (CBT) program designed to improve children's emotional health. Each child has their own workbook and group leaders have a comprehensive manual specifying key learning points, objectives, and activities for each session. The intervention trialed in this study involves nine, 60-minute weekly sessions delivered to whole classes of children. Through a range of age-appropriate fun activities including stories, quizzes, role plays and games, children learn practical skills to control their anxiety. They are helped to identify their feelings and to learn to relax, thereby managing their anxious feelings. They are helped to identify unhelpful thoughts (anxiety increasing) and to replace them with more helpful (anxiety reducing) thoughts. Finally, they are helped to face and overcome their problems and challenges rather than avoid them. Written work is kept to a minimum and each session uses a variety of different materials and activities to engage and maintain the interest of the children. Initial training and regular supervision of class leaders will be provided by an accredited FRIENDS trainer.

An additional session for parents/carers is conducted to provide parents with an overview of the program, the CBT rationale, and the skills the children will learn. In addition, parents receive a summary sheet detailing the key learning points of each session and the ideas their child will be practicing so that they can reinforce and encourage their use at home.

### Study arms

Forty-one schools were randomized to one of the three conditions; school-led FRIENDS, health-led FRIENDS or treatment as usual (control). One school in the control arm withdrew after randomization.

### School-led friends

Each participating school will be asked to identify school staff (class teachers, special educational needs coordinators and/or teaching assistants) to deliver FRIENDS. These staff members will attend a two-day training event to familiarize them with the nature, extent and presentation of anxiety and depression in children and the CBT model. They will work through each of the FRIENDS sessions and have opportunities to practice the exercises and familiarize themselves with the materials and key learning points.

At each session the school staff will be assisted by two health professionals external to the school, to ensure that at least three adults are present in the class during the session delivery. The health professionals will not be responsible for leading the session but for supporting the school leader. During delivery of the program, biweekly supervision groups will be established to review session aims and content and address any problems with implementation.

### Health-led friends

This condition will be delivered by health professionals (Band 6, for example, school nurses, psychology assistants) external to the school (with the class teacher providing required assistance). These are not mental health specialists but health professionals with a lower level of training or expertise. They will receive the same initial training as specified above and will attend a biweekly supervision group.

### Treatment as usual

In this group, children will participate in the usual PSHE sessions provided by the school. These sessions will be planned and led by the class teacher.

In order to more specifically define Personal Social and Health Education (PSHE) within each school the head teacher, school PSHE coordinator and/or the year 5 class teacher will participate in a semi-structured interview. This interview will be undertaken at the end of the school term and will assess whether the school is following the national curriculum, what additional interventions might be running in the school and their content. The interview will determine the PSHE topics covered by the 9- and 10-year-old children during the study period, the way they are addressed (dedicated sessions, integration, circle time, etcetera), the length of time devoted to the PSHE curriculum and the number of adults (for example, teachers, assistants, volunteers, trainees) in the classroom.

### Outcome measures

All child outcome data are collected by self-completed questionnaires administered by researchers blind to the child’s trial allocation. Questionnaires are completed at school in classes at baseline, at 6 months and at 12 months.

### Primary outcome measures

The primary outcome measure notes changes in the level of symptoms on the Revised Child Anxiety and Depression Scale (RCADS) [23]. This is a recent modification of the Spence Children’s Anxiety Scale [24], which was revised to correspond more closely to DSM-IV criteria for anxiety and depression [25]. The 30-item scale (RCADS-30) assesses anxiety in the areas of social phobia, separation anxiety, obsessive compulsive disorder, panic disorder and generalized anxiety disorder and also assesses major depressive disorder. The RCADS-30 has good internal consistency, test-re-test stability and good convergent and divergent validity [26,27].

### Secondary outcomes

Children will complete the Rosenberg Self-Esteem Scale to assess changes in self-worth and acceptance [28]. The Penn State Worry Questionnaire for Children [29] assesses the tendency of children to engage in excessive, generalized and uncontrolled worry. The degree to which children have bullied others or have been the victim of bullying, satisfaction with six aspects of everyday life (school, appearance, family, home, friendships and health), and overall life satisfaction (subjective well-being) will be assessed.

Parents will be asked to complete the Strength and Difficulties Questionnaire (SDQ), a brief, widely used behavioral screening questionnaire that assesses emotional symptoms, conduct problems, hyperactivity and/or inattention, peer relationship problems, and prosocial behavior [30,31]. The Parent completed Revised Child Anxiety and Depression Scale (RCADS-30-P) is a 30-item parent version of the primary outcome measure completed by children. The RCADS-30-P has high internal consistency, test-re-test reliability and good convergent and divergent validity [32].

Class teachers will be asked to complete the impact rating of the Strengths and Difficulties Questionnaire (SDQ) for all children in their class. This assesses the teacher perception of whether a child has a problem, and if so, enquires about chronicity, distress, social impairment and burden.

### Outcomes for the economic evaluation

In order to assess cost-utility, all children will complete the Child Health Utility-9D (CHU-9D) at baseline and at 6 and 12 months [33]. The CHU-9D, a validated measure of pediatric health-related quality of life, is short (nine items) and has been specifically developed for use with children aged 7 to 11 years. The use of the CHU-9D will allow us to assess how improvements in mental health (anxiety and depression) translate into changes in overall health-related quality of life.

For collecting health, social care and educational resource use data, structured interviews will be conducted with a sample of carers/parents for each of 300 children, with approximately 100 children from each of the three conditions. All parents will be invited to participate and they will be offered £20 to cover the cost of their time. They will complete the Client Receipt of Services Questionnaire, a structured interview that assesses their child’s use of social, health and educational services over the past 6 months [34]. In addition, a screen of parental health and mental health, assessment of life events and a survey of child leisure and sport activities will be undertaken.

### Power calculation

The study was powered to detect a difference between FRIENDS (Health- and School-led) and usual PSHE. Based on an intra-cluster correlation coefficient (ICC) of 0.02, 28 pupils per class, 90% consent and 80% retention, effect sizes in the range of 0.28 to 0.30 SDs are detectable with 80% power and 5% two-sided alpha with 45 to 54 schools (that is, 1,134 to 1,360 consenting pupils). A standardized treatment effect size of 0.3 is equivalent to an estimated difference on the RCADS-30 of 3.6 points based on an SD of 12.

### Statistical analysis

Data analysis will be undertaken blinded to allocation. Analysis and presentation of data will be in accordance with Consolidated Standards of Reporting Trials (CONSORT) guidelines and in particular the extension to cluster randomized trials [35]. The primary comparative analyses will be conducted on an intention-to-treat (ITT) basis with due emphasis placed on confidence intervals for the between-arm comparisons. Descriptive student- and class-level statistics will be used to ascertain any marked imbalance between the arms at baseline.

The primary analysis will employ a mixed effect linear regression model to compare the active CBT interventions (health- and school-led) versus treatment, adjusting as usual for stratification variables and baseline score, and taking appropriate account of the hierarchical nature of the data (repeated measures, children, classes and schools). Sensitivity analyses making different assumptions will be conducted to investigate the potential effects of missing data.

The extent of missing data will be reported and baseline factors will be compared for completers and non-completers to assess the extent of any bias that may result. Analysis will be undertaken using intention to treat (ITT). The potential for bias, dependent on the extent of missing data, will be assessed. Using the framework specified by White *et al.* an appropriate approach will be used to impute missing values in both outcome and descriptor variables [36]. A further complete case analysis may also be undertaken and compared to the intention to treat results.

Secondary analyses will include: 1) repeating the primary analysis adjusting for any variables exhibiting marked imbalance at baseline to examine whether this influences the findings; 2) comparison of children who score high (that is, top 10% of total RCADS scores in each trial arm) and low on anxiety and mood at baseline (that is, remaining 90%) to examine who most benefits from these interventions; 3) similar analyses for other secondary outcomes (using appropriate multi-level models and adjusting *P* values for multiple testing); 4) investigation of process measures such as number of sessions attended; and 5) investigation of possible treatment moderators (for example, gender) and mediators (for example, school pedagogic orientation). Finally, although the study is not sufficiently powered to detect a difference between the two FRIENDS arms (school- and health-led) an exploratory analysis will be undertaken.

### Economic evaluation

The analysis of the cost data, the CHU-9D data and cost-effectiveness analysis will be conducted according to current best practice methods for conducting economic evaluation alongside trials, [37,38] and specifically alongside cluster-randomized controlled trials [39]. Incremental costs, incremental effects, and where relevant incremental cost-effectiveness ratios (ICERs) will be estimated, comparing the three conditions. The incremental cost per unit quality-adjusted life year (QALY) increase will be estimated. Both unadjusted analyses and adjusted analyses will be carried out, adjusting for site, mode of delivery, number of students and number of classes.

Random effects bivariate linear regression models will be fitted to model cost and effectiveness (QALY) simultaneously, allowing for correlation within clusters and correlation between cost and effectiveness score within participants [40]. From these models we will estimate: the mean difference in costs of the three conditions and corresponding standard error; the mean difference in effect and corresponding standard error; and, indirectly via the variance-covariance matrix of the regression coefficients, the correlation between the mean cost difference and the mean effect difference. Where the interventions are estimated to have both higher costs and greater effectiveness than control, we will use Fieller’s method to obtain a parametric estimate of the 95% confidence interval for the ICER and to construct the cost-effectiveness acceptability curve (CEAC). Both parametric and non-parametric bootstrap estimates of the confidence interval for the ICERs will be estimated.

### Study status

The trial started in January 2011 and 41 schools were randomized although one subsequently withdrew. Baseline assessments and intervention delivery started in September 2011 and twelve-month assessments are scheduled to be completed by May 2013.

## Abbreviations

CEAC: Cost-effectiveness acceptability curve; CONSORT: Consolidated Standards of Reporting Trials; ICC: Intra-cluster correlation coefficient; ICERs: Incremental cost-effectiveness ratios; ITT: Intention-to-treat; PACES: Preventing Anxiety in Children through Education in Schools; QALY: Quality-adjusted life years; RCADS-30-P: Parent completed Revised Child Anxiety and Depression Scale; RCADS: Revised Child Anxiety and Depression Scale; RCT: Randomized controlled trial; SDQ: Strengths and Difficulties Questionnaire.

## Competing interests

None of the authors have any competing interests arising from this research.

## Authors’ contributions

PS, GT, RA, NS and RB conceived the study and led the bid to secure funding for this work. They have contributed to the development of the protocol and are involved in managing and advising on the project. ES is the trial manager and has contributed to the development of the protocol and the drafting of this paper. All authors read and approved the final manuscript.
